# Pretarsal orbicularis oculi muscle tightening with skin flap excision in the treatment of lower eyelid involutional entropion

**DOI:** 10.1186/s12886-021-02214-9

**Published:** 2021-12-15

**Authors:** Jianhao Cai, Yuansheng Zhou, Wenjuan Lv, Wenxia Chen, Weihao Cai, Tsz Kin Ng, Zeyi Li

**Affiliations:** 1 Joint Shantou International Eye Centre of Shantou University and The Chinese University of Hong Kong, North Dongxia Road, Shantou, 515041 Guangdong China; 2 Shantou University Medical College, Shantou, 515041 Guangdong China; 3 Department of Ophthalmology and Visual Sciences, The Chinese University of Hong Kong, Kowloon, Hong Kong

**Keywords:** Involutional entropion, Surgical treatment, Orbicularis oculi muscle, Skin flap excision

## Abstract

**Background:**

To evaluate a modified technique for involutional entropion correction in a retrospective cohort study.

**Methods:**

The patients with involutional entropion eyelid were corrected by tightening the pretarsal orbicularis oculi muscle and excising the excess skin of the lower eyelid. The patients received correction surgery from April 2013 to March 2019 were followed up for more than 6 months postoperatively. The outcome measures included the complications and the recurrence rates.

**Results:**

Total 152 patients (169 eyes) were included. The mean follow-up period was 29.6 months (range: 6–36 months). Postoperative ectropion (over-correction) was observed in 1 patient with 1 eyelid (0.59%); yet, no further surgery was needed for this patient. Recurrence of entropion was found in 1 patient (0.59%). The patient with recurrent entropion received repeated surgery with the same method and achieved a good eyelid position.

**Conclusions:**

This study demonstrated that tightening the pretarsal orbicularis oculi muscle and excising the excess skin of the lower eyelid could be an effective surgical method to correct lower eyelid involutional entropion. This method is technically easy with a low recurrence rate and not associated with significant complications in Asians.

## Background

Involutional entropion is a condition with eyelid malposition, mainly affecting the lower eyelid, and causes ocular discomfort, epiphora, foreign body sensation and corneal ulceration. The prevalence of involutional entropion among the elderly population is 2.1% and increases with age [1]. Higher incidences were reported among East Asians as compared to non-East-Asians [2]. Significant horizontal eyelid laxity was demonstrated in 79% of patients with primary and recurrent involutional entropion [3]. Etiology of involutional entropion includes horizontal lid laxity, vertical lid instability, over-riding of the pretarsal by the preseptal orbicularis muscle and orbital septum laxity [3, 4]. Vertical lid instability mainly results from attenuation, dehiscence or disinsertion of the lower lid retractors [3]. These causes of entropion could be corrected surgically.

Various surgical approaches aim to correct the underlying problems causing entropion, including the Wies procedure, Quickert sutures, lateral tarsal strip procedure, and lower lid retractor reinsertion [5]. There is no established standard criteria in terms of the choice of the surgical method. Wheeler, in 1938, described an operation for the correction of spastic entropion that a band of orbicularis muscle from the anterior surface of the tarsal plate was dissected, overlapped, and reattached to the tarso-orbital fascia just below the tarsus [6]. The Wheeler’s procedure was subsequently modified by many different surgeons. Based on the Wheeler’s procedure, Hill et al. described a combination of techniques that consisted of the following: (1) curettage; (2) suturing of the upper edge of the skin incision to the lower border of the tarsus; and (3) suturing a strip of the orbicularis muscle to the lower tarsal plate border. In addition, the lateral lower eyelid was shortened by a full-thickness excision [7].

In this study, we further modified the Wheeler’s and Hill’s procedures and evaluated the outcomes of the modified procedures by fixating a strip of shortened orbicularis muscle to the lower tarsal plate border and excising the excessive skin for involutional entropion without lower eyelid laxity.

## Material and methods

### Participants

This retrospective study included 152 consecutive patients (169 eyelids) with involutional lower eyelid entropion surgery performed by one ocular plastic surgeon (J.C.) from April 2013 to March 2019. The inclusion criteria was based on the diagnosis of involutional lower eyelid entropion characterized by the inward turn of the lower eyelid, and a history of symptoms of ocular irritation, such as eye rubbing, epiphora and photophobia. The patients with any other etiology of entropion, such as congenital, cicatricial, paralytic or spasmodic entropion, were not included. This study only included the patients undergoing orbicularis muscle shortening surgery without lateral tarsal strip. The pinch test, which the central aspect of the lower lid skin was grasped and the lower eyelid was pulled away from the globe, was performed to exclude the patients with eyelid laxity. The distance between the globe and the posterior aspect of the lower lid was measured. The pinch test with distance > 8 mm was considered as eyelid laxity [1]. The study was firmly adhered to the tenets of the Helsinki declaration and written informed consent was obtained from every patient.

All patients have been followed up for at least more than 6 months postoperatively. The surgical outcome was considered as successful when the eyelid margin did not turn towards the globe at rest, and no eyelashes touched the globe during the follow-up visit. Conversely, recurrence was defined as the eyelid margin did turn toward the globe at rest, or the eyelashes did touch the globe.

### Surgical techniques

**Fig. 1:**
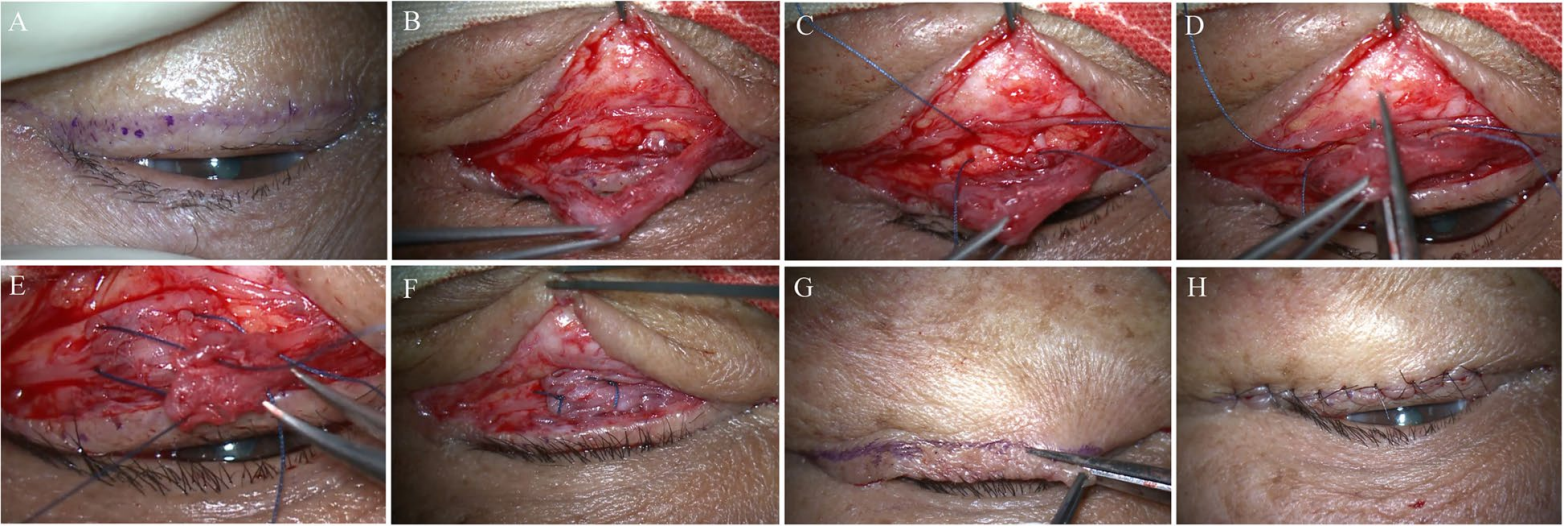
Surgical steps. **A** Skin incision 2 mm below the lower eyelashes. **B** The orbicularis oculi muscle was separated from the orbital septum. **C** Two double-arm sutures were preset in the inferior tarsal border. **D** The strip of orbicularis muscle was cut into two halves vertically. **E** Two double-arm sutures passed through the orbicularis muscles in sequence. **F** The two sutures were tied and the orbicularis oculi muscle was shortened and fixed onto the inferior tarsal border. **G** A redundant skin flap was excised. **H** The immediate postoperative effect.

The procedure was performed under local anesthesia for all patients. The lower eyelid was infiltrated with 2 mL of 2% lidocaine containing 1:100,000 epinephrine. A linear skin incision was made at 2 mm below the lower eyelashes and then extended to about 5 mm laterally and inferiorly along the crow’s feet (Fig. 1A). Dissection was firstly performed subcutaneously until near the inferior orbital rim and then between the tarsus and orbicularis muscle. A strip of the orbicularis muscle was separated and identified, and cut into two halves vertically (Fig. 1B). Two double-arm 6–0 Vicryl® sutures were preset in the inferior tarsal border (Fig. 1C). The strip of the orbicularis muscle was cut into two halves vertically (Fig. 1D). The two tips of orbicularis strips were overlapped maximally wherever possible for shortening, but without malposition or twisting of the eyelid margins. Two double-arm 6–0 Vicryl® sutures passed through the orbicularis muscles in sequence and were tied in a slipknot (Fig. 1E). A confirmation test was performed by asking the patients to close their eyes tightly. The eyelid was expected to stay stably. If the lower eyelid turned inward after the eyes closing, it implied that the surgery was undercorrected. Otherwise, if the lower eyelid became distorted, it suggested overcorrection. In these situations, the two sutures need to be replaced. If the lower eyelid kept stable after closing the eyes, the slipknot was retied (Fig. 1F). A redundant skin flap was excised (Fig. 1G). Finally, the skin was closed with an interrupted or running suture (Fig. 1H). Antibiotic eye ointment was instilled in the inferior conjunctival fornix and the wound. The first postoperative visit was 1 week after the surgery, and the suture was removed at the first postoperative follow-up visit.

## Results

In total, 152 patients with169 studied eyes were enrolled in the study. The mean age of the patients at surgery was 70.66 years old (range: 43–94 years old). Among them, 69 eyelids were from 66 men, and 100 eyelids from 86 women. Seventeen patients were bilateral and 135 were unilateral.

**Fig. 2:**
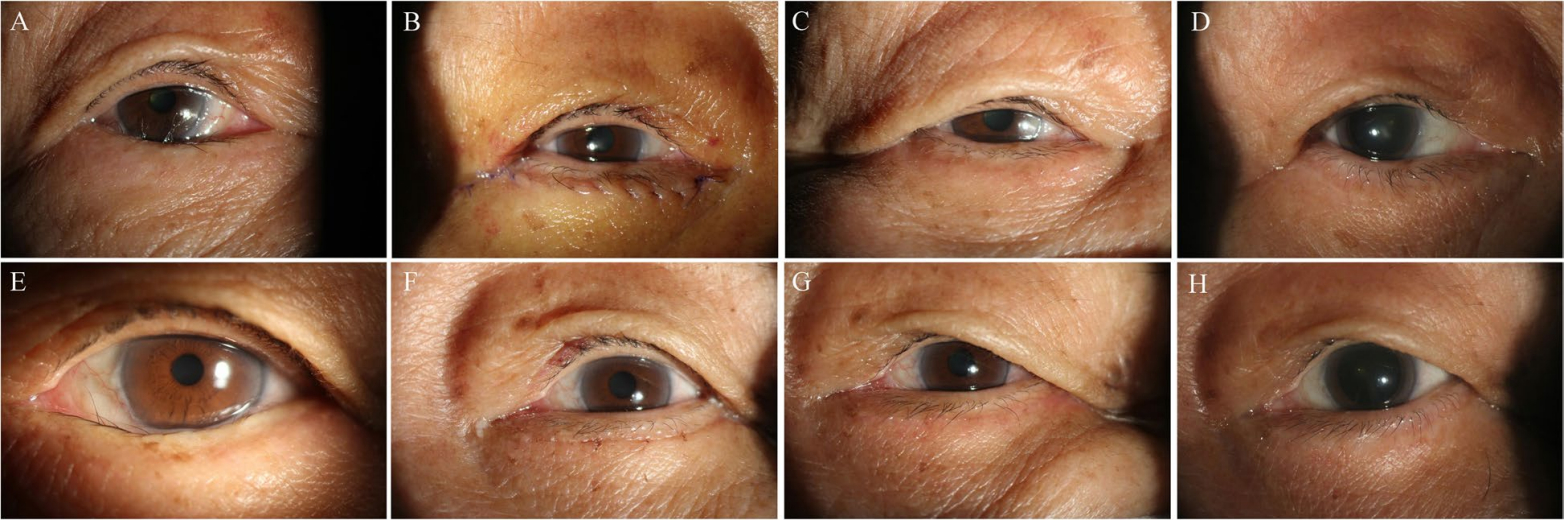
Photographs of a female subject diagnosed with involutional entropion and treated by pretarsal orbicularis oculi muscle tightening with skin flap excision. **A** Photograph taken preoperatively (OD). **B** 1 week after the surgery, before removing the sutures (OD). **C** 1 month after the surgery (OD). **D** 9 months after the surgery (OD). **E** Photograph taken preoperatively (OS). **F** Sutures were removed 1 week after surgery (OS). **G** 1 month after the surgery (OS). **H** 9 months after the surgery (OS).

Fig. 2 showed a female patient, who was diagnosed with involutional entropion in both eyes, was treated by pretarsal orbicularis oculi muscle tightening with skin flap excision. The right eye surgery was performed 1 week after the left eye surgery.

The surgical outcomes of 152 patients were monitored over a mean follow-up period of 29.6 months (range: 6–36 months). During the follow-up period, one patient showed overcorrection, and he had a severe lower eyelid pouch. The overcorrection was mild, and the patient was asymptomatic; therefore, no further surgery was performed.

Recurrence of entropion was found in 1 patient (0.59%). The patient with recurrent entropion received repeated surgery with the same method and achieved a good eyelid position. In the second surgery, we found that the orbicularis muscle fixed in the first surgery moved forward onto the centroid of the lower tarsus, which could be the cause of the recurrence. No other significant complications were observed. The potential risks include hair follicles damage, excessive bleeding and infection.

## Discussion

Involutional entropion is a frequent eyelid malposition that could threaten the eyesight. It is more common in Asian patients than non-Asian patients. Previous studies have identified differences between the lower eyelids of Asians and non-Asians based on gross dissection and microscopic examination [5, 8]. Asian individuals tend to have puffier, filled lower eyelids and absent or indistinct lid creases. Moreover, lower eyelid retractor fusional location with the orbital septum was higher in Asians. In addition, lower eyelid retractors had indistinct junctions and no fusion with orbital septum in Asians. Horizontal lid laxity was also less common in Asians [5, 8–10]. The anatomical differences between Asian and non-Asian patients could elucidate the pathogenesis of Involutional entropion.

A variety of surgical options for involutional entropion exist, and there is no established standard criteria. For the involutional entropion correction surgery, recurrence is the most critical complication [11, 12]. An ideally effective technique should be able to correct all causative factors while minimizing the recurrence rate. Quickert sutures could mechanically tighten the lower lid retractors without the need for a skin incision [13]. Quickert sutures are easy to perform and known to be a safe and fast treatment for involutional entropion, but with a high rate of recurrence among Asian patients. Jang et al. reported a rate of recurrence (49.3%) for the Quickert sutures treatment on involutional entropion in Korean during the 2-year follow-up period [14]. The lateral tarsal strip is a popular procedure for eyelid shortening and could be used either in entropion or ectropion correction. Although lateral tarsal strip is effective in tightening the eyelid, it could only correct the lower lid laxity conditions; yet, it would cause undercorrection, webbing, recurrence and disruption of the lateral canthal angle [15, 16].

According to the anatomical characteristics of the patients and the etiology, the key point of success should be choosing an appropriate surgical method. Orbicularis muscle overriding plays a key role in the development of Asiatic involutional entropion [17]. Valencia et al. studied microscopic changes of the cadaver eyelids, and found that the loose attachment of the preseptal orbicularis oculi muscle on the orbital septum could contribute to the development of involutional entropion [18]. The Wheeler’s procedure and Hill’s procedure are both suitable to remove the overriding orbicularis muscle. Herein, in our study, we modified the Wheeler’s procedure and the simplified Hill’s procedure for involutional entropion without documented eyelid laxity in the Chinese population. Compared to the traditional Whleer’s and Hill’s procedures, our modified techniques included that: 1) The fixed position was on the lower edge of the tarsal plate, not on the tarso-orbital fascia so that the fixed orbicularis muscle was not easy to slip off. The procedures of curettage and suturing of the upper edge of the skin incision to the lower border of the tarsus were removed in order to improve efficiency and avoid skin folds on lower eyelid when eyes were looking down. The tarsus acts like a seesaw. When the orbicularis muscle was pressed against its upper edge, it tended to become inverted. When the orbicularis muscle was pressed down on its lower edge, it was more prone to evert, especially when it contracted. We shortened the orbicularis muscle at the same time in order to enhance its pressing effect on the lower edge of the tarsus when it contracted. On the other hand, shortening the orbicularis muscle also contributed to the enhancement of eyelid horizontal maintenance strength. 2) The use of two double-needle sutures could make the orbicularis muscle fixed more firmly. At the same time, it was helpful to timely adjust the shortening of the orbicularis muscle according to the condition of the eyelid margin during the operation. 3) Removal of part of the lower eyelid skin makes the lower eyelid skin incision smoother, and, at the same time, assist in the correction of lower eyelid inversion although it does not play a key role in this operation. Compared to Quickert sutures and the lateral tarsal strip, our modified procedures showed a low recurrence rate of 0.59% in our Chinese patients.

Our modified procedure presented in this study is suitable for lower eyelid involutional entropion without horizontal relaxation. Overcorrection could easily happen when the patient had a significant horizontal relaxation (a pinch test of > 8 mm) and severe lower eyelid pouch preoperatively. Some methods could avoid the postoperative overcorrection. Precise preoperative assessment should be performed to exclude horizontal relaxation. Intraoperatively, the position of the palpebral margin should be assessed after refixation of the orbicularis muscle strips. During the operation, the patients were forced to close their eyes, and the lower eyelid margin was observed to evaluate the surgical effect. If overcorrection was presented, a horizontal shortening procedure was strongly recommended, such as the lateral tarsal strip sling. If the patient had obvious bags under the eyes, it was also recommended to remove the bags to avoid overcorrection after the surgery. No lower lid retraction was found with our technique in this study. The fixation point of the tightening orbicularis muscle was the lower margin of the tarsus. The orbicularis muscle likes a ligament supporting the tarsus. When it contracted, it became straight and could hold the tarsus upward. Therefore, it did not cause lower eyelid retraction, but could help to improve it.

The amount of redundant skin excised should be minimized. The main factor to correct the inverted eyelid margin was the refixation of the orbicularis muscle but not the traction of lower eyelid skin. Overcorrection could be a complication when excessive skin was removed. In our study, we asked the patient to look upward and gently draped the lower eyelid skin overlying the lid margin, then marked and excised to smooth the skin incision.

## Conclusions

This study demonstrated that pretarsal orbicularis oculi muscle tightening with skin flap excision was highly effective for the correction of involutional lower eyelid entropion. Although this approach does not correct lateral canthal tendon laxity, it does solve the overriding of orbicularis muscle and correct the lower eyelid position. A recurrence rate of 0.59% was noted in our procedure. Pretarsal orbicularis oculi muscle tightening with skin flap excision should be a safe and effective method for involutional lower eyelid entropion.

## Data Availability

The datasets used and analyzed during the current study are available from the corresponding authors on reasonable request.

## Abbreviations

OD: Right eye
OS: Left eye
